# Editorial: Advance in diagnostics for central nervous system infection

**DOI:** 10.3389/fneur.2023.1200056

**Published:** 2023-04-25

**Authors:** Samir Abu-Rumeileh, Ying Luo

**Affiliations:** ^1^Department of Neurology, Martin-Luther-University Halle-Wittenberg, Halle, Germany; ^2^Department of Immunology, UT Southwestern Medical Center Dallas, Dallas, TX, United States

**Keywords:** neuroinfectious disease, cerebrospinal fluid, biomarkers, meningitis, encephalitis

The Research Topic “*Advance in Diagnostics for Central Nervous System Infection*” aims to collect studies that focus on recent advances and discoveries in the diagnosis of neuroinfectious diseases. There is an urgent need to optimize the diagnostic assessment of these disorders due to the high disease burden, the elevated incidence of long-term sequelae, and the importance of rapid therapeutical decisions in terms of prognosis. To date, most conventional diagnostic methods are suboptimal in terms of sensitivity and specificity, probably due to the complex interplay between the central nervous system and pathogens. Nevertheless, the introduction of new microbiological tests, such as novel PCR techniques and proteomic approaches, has shown promising results. In this context, the recent COVID-19 pandemic has prompted worldwide healthcare systems to reconsider the management of infectious diseases as a priority (1). Of note, given the possible limited accessibility for imaging investigations during pandemic-imposed restrictions, the search for easily measurable biofluid markers, with the aim of overcoming the limitation associated with medical isolation procedures, has been of interest (1). Here, we report on the most important insights from our collection.

The study by Si et al. encapsulates many of the above-mentioned topics. Indeed, the authors tested a novel multiplex PCR panel, which can simultaneously and rapidly detect 18 common meningoencephalitis (ME) pathogens, in cerebrospinal fluid (CSF) samples obtained from 139 patients with suspected central nervous system infection. The new technique showed promising results, especially in the detection of poly-infections, and good consistency (89.5%) with respect to conventional testing for the diagnosis of *Mycobacterium tuberculosis*. However, in 40% of cases, the pathogens were correctly diagnosed by conventional testing only, suggesting the need for further validation studies for the new method in larger cohorts. Nevertheless, it might represent a new potential tool, besides conventional testing and clinical assessment, for the early diagnosis of neuroinfectious diseases.

Similarly, Beaver et al. focused their study on CSF shunt infection, the diagnosis of which is still hampered by the low accuracy of bacterial cultures and CSF indices. The authors performed a proteomic study in serial CSF samples obtained from an animal model with a central nervous system (CNS) catheter infection and from another with a sterile catheter placement. The aim was to assess possible shared and distinct CSF proteomic changes during *S. epidermidis, C. acnes, and P. aeruginosa* shunt infection over time. Although the number of differentially expressed proteins was variable across the three bacterial species, the authors found a shared proteome across diverse pathogens, especially at early points, which was also unique compared with the sterile injury response. If future studies confirm these findings, the identification of shared candidate CSF biomarkers across distinct bacterial infections species may represent a step forward for the early diagnosis and management of these diseases.

Cryptococcal antigen testing has shown a very high performance in diagnosing cryptococcal disease, especially in HIV patients (2). Moreover, other CSF and blood markers (i.e. chemokine ligand 13 and neurofilament light chain protein) have been investigated recently (3). Nevertheless, diagnostic errors and delays might still occur and be associated with worse neurological outcomes and increased mortality. The study by Wen et al. investigated a cohort of patients with cryptococcal meningitis and reported a misdiagnosis at the initial visit in 37 out of 98 included cases (37.8%). The authors suggested that this could be related to the high proportion of non-HIV underlying conditions (e.g., rheumatological diseases, history of corticosteroid use, etc), in which cultures and antigen tests might perform worse due to the relatively lower cryptococcus load. Furthermore, the authors assessed the short-term and long-term outcomes (more than 1 year) of ventriculoperitoneal shunt placement (VPS) in the same cohort. All patients received standard antifungal therapy and 15 underwent additional VPS. The study showed that critically ill patients treated with VPS showed better overall survival, even if the long-term Barthel Index did not differ between the subgroups receiving surgical or conservative treatment.

Acute encephalopathy with biphasic seizures and late reduced diffusion (AESD) is a severe neurological complication of infantile febrile infectious diseases (e.g., influenza and exanthem subitem) and has a high incidence in Japan. Proinflammatory pathways including interleukin 1 beta (*IL-1*β) seem to play a role in the pathogenesis of febrile seizures (FS), a disease that may clinically overlap with AESD. Most interestingly, Asian patients with febrile seizures (FS) showed a significant excess in the TT genotype of the *IL-1*β rs16944 polymorphism, which is usually associated with increased *IL-1*β expression. To investigate these findings in AESD, Shibata et al. performed a genetic association study of rs16944 in 283 AESD patients and found a significantly lower proportion of the TT genotype compared with a control group (i.e., 104 healthy Japanese adults with available genotyping data), suggesting that AESD and FS did not share the same genetic background.

## Conclusion

In conclusion, all the articles included in the Research Topic, although heterogeneous in their approach, provided novel insights into the diagnosis of neuroinfectious diseases and showed the potential to ameliorate their management by accelerating the diagnostic assessment and therapeutic intervention in different clinical settings.

## Author contributions

SA-R wrote the manuscript. YL reviewed the manuscript and approved the final version. All authors contributed to the article and approved the submitted version.
